# The assembly of integrated rat intestinal‐hepatocyte cultures

**DOI:** 10.1002/btm2.10146

**Published:** 2019-11-09

**Authors:** Anjaney Kothari, Padmavathy Rajagopalan

**Affiliations:** ^1^ School of Biomedical Engineering and Sciences Virginia Tech Blacksburg Virginia; ^2^ Department of Chemical Engineering Virginia Tech Blacksburg Virginia; ^3^ ICTAS Center for Systems Biology of Engineered Tissues Virginia Tech Blacksburg Virginia

**Keywords:** integrated cultures, liver, small intestine

## Abstract

The jejunum is the segment of the small intestine responsible for several metabolism and biotransformation functions. In this report, we have cultured rat jejunum explants *in vitro* and integrated them with hepatocyte cultures. We have also investigated the changes in jejunum function at different locations since spatial variations in intestinal functions have been reported previously. We divided the length of the rat jejunum into three distinct regions of approximately 9 cm each. We defined the regions as proximal (adjacent to the duodenum), medial, and distal (adjacent to the ileum). Spatiotemporal variations in functions were observed between these regions within the jejunum. Alkaline phosphatase activity (a marker of enterocyte function), decreased twofold between the proximal and distal regions at 4 hr. Lysozyme activity (a marker of Paneth cell function) increased from the proximal to the distal jejunum by 40% at 24 hr. Mucin‐covered areas, a marker of goblet cell function, increased by twofold between the proximal and distal segments of the jejunum at 24 hr. When hepatocytes were integrated with proximal jejunum explants, statistically higher urea (~2.4‐fold) and mucin (57%) production were observed in the jejunum explants. The integrated intestine‐liver cultures can be used as a platform for future investigations.

## 1 INTRODUCTION

The small intestine is a complex organ that conducts a wide range of functions including the digestion of food, absorption of nutrients, and metabolism of drugs and toxicants.^1^, ^2^, ^3^ It is divided into three distinct segments, namely the duodenum, the jejunum, and the ileum, each of which is responsible for different specialized functions.^1^, ^4^ The intestine is also divided into different layers radially, namely the mucosa, the submucosa, the muscularis externa, and the serosa/adventitia.^1^, ^4^ The mucosa forms the intestinal lumen where it absorbs nutrients and secretes proteins.^1^, ^4^ The epithelial layer of the intestinal mucosa is composed of five primary cell types: enterocytes (>90% of the epithelial cell population), goblet cells (~8–10%), enteroendocrine cells (<1%), Paneth cells, and stem cells.^2^, ^4^ The first three of these cell types are arranged along finger‐like projections known as villi.^1^, ^2^ Enterocytes are the primary absorptive cells that also express biotransformation enzymes such as cytochrome P450s (such as CYP2E1) and alcohol dehydrogenase (ADH).^1^, ^3^, ^5^ Goblet cells are involved in the secretion of glycoproteins called mucins that constitute the mucus barrier in the intestine, while enteroendocrine cells are responsible for the secretion of hormones such as secretin and serotonin.^1^, ^2^ The villi are accompanied by corresponding invaginations in the mucosa called the Crypts of Lieberkühn.^1^, ^2^ These are populated by Paneth cells that secrete antimicrobial proteins including lysozyme and defensins, and stem cells that renew the mucosal epithelium.^1^, ^2^

The small intestine and the liver work together to modulate several physiological functions such as, bile acid homeostasis, urea cycle, and absorption and metabolism of orally administered drugs and toxicants.^4^, ^6^ Disruption of the small intestine‐liver axis is implicated in diseases such as alcoholic liver disease and hepatic encephalopathy.^7^ The interactions between these two organs highlight the need to study them together.

*In vitro* culture of the small intestine has been conducted through several means such as explants, primary epithelial cells, immortalized cell lines, and more recently, organoids.^4^, ^8^, ^9^ Intestinal explants are comprised of all cell types in the epithelium as well as the underlying layers such as the submucosal mesenchyme.^9^, ^10^ These explants have been used to investigate a wide range of intestinal phenomena.^4^, ^8^, ^11^ The adult small intestine has only been cultured for shorter time periods typically ranging from 4 hr to 5 days.^4^, ^8^, ^11^ Explants from the adult rat jejunum have been shown to degrade after 24 hr in culture.^8^

*In vitro* studies on integrating the intestine and the liver have focused on using cell lines such as Caco‐2 cells with either primary hepatocytes or hepatocyte cell lines such as HepG2 cells.^4^ So far, there has only been one study where primary models of the intestine and liver were integrated.^12^ Van Midwoud et al.^12^ integrated rat precision‐cut intestinal slices (PCIS) and precision‐cut liver slices (PCLS) to investigate the induction of Phase I and Phase II enzymes and maintenance of bile acid homeostasis over a 7 hr period.^12^ This study did not investigate the effects of location or integration on individual functions of the PCIS and PCLS.

Among the duodenum, jejunum, and ileum, the jejunum is critical for the digestion of food as well as the absorption of nutrients such as sugars, fats, and amino acids.^1^, ^13^ The jejunum has the highest surface area for absorption among the three segments.^14^, ^15^ It is primarily responsible for the absorption of bile salts transported from the liver into the intestine.^16^, ^17^ In addition, the jejunum has the highest abundance of several Phase I and Phase II drug‐metabolizing enzymes in the small intestine.^3^ Therefore, we have focused our investigations on the jejunum.

*In vivo* and *ex vivo* studies on the intestine have revealed several spatial trends in its properties.^5^, ^18^, ^19^ For example, activities of enzymes such as ALP and ADH decrease,^5^, ^18^ while lysozyme activity and the thickness of the mucus barrier increase along the length of the small intestine.^18^, ^19^ However, investigations of location‐dependent trends have been reported primarily through in vivo or ex vivo studies.^5^, ^18^, ^19^ Moreover, these reports have focused on the differences among the duodenum, the jejunum, and the ileum. There are a few ex vivo reports where the entire length of the small intestine was divided into small sections to study spatial variations.^20^, ^21^ To the best of our knowledge, there are no studies that have reported changes within the jejunum.

Since the location of intestinal cells plays a crucial role in determining their function,^5^, ^18^, ^19^ investigating the jejunum as a whole does not allow for the delineation of these spatial effects. Hence isolating the effects of location on the functions of the jejunum in vitro necessitates its division into short segments obtained from different locations. We report the culture of rat jejunum explants to investigate location‐dependent variations in biochemical markers of enterocytes, Paneth, and goblet cells. In this report, we have divided the length of the rat jejunum into three regions; proximal (adjacent to the duodenum, ~9 cm), medial (~9 cm), and distal (adjacent to the ileum, ~9 cm). Jejunum explants were integrated with primary rat hepatocytes in collagen sandwich (CS) cultures. The effects of integration were evaluated through monitoring changes in intestinal cells and hepatocytes.

## 2 RESULTS

### 2.1 Identification of different segments of the rat small intestine

**Figure 1:**
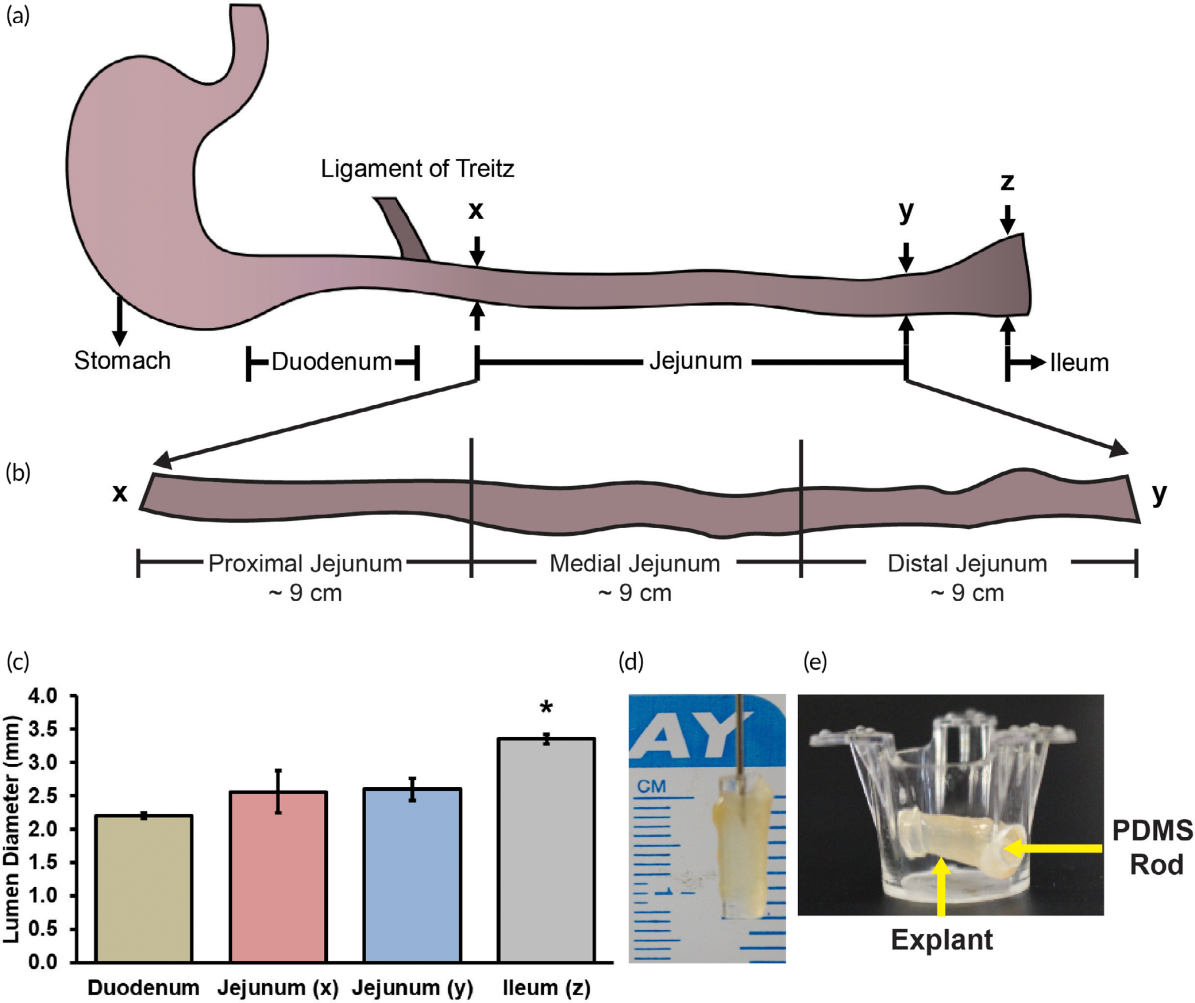
(a) Schematic of the small intestine showing its longitudinal segments. The duodenum ends at the ligament of Treitz. “**x**” and “**y**” denote the start and the end of the jejunum, respectively. “**z**” denotes the start of the ileum. (b) The jejunum was divided into three segments, namely proximal, medial, and distal jejunum. (c) The jejunum–ileum transition is marked by an increase in diameter of the intestine. (d) To excise explants, the jejunum was inverted, pulled up on a polydimethylsiloxane (PDMS) rod and cut at a length of ~10 mm. (e) The PDMS rod was suspended in a Transwell® insert with two diametrically opposite holes drilled through it. * denotes *p* ≤ .05 relative to duodenum, jejunum (*x*), and jejunum (*y*)

The total length of the small intestine excised from the rats was found to be 92.7 ± 3.8 cm (*n* = 7 intestines). The duodenum, jejunum, and ileum were identified and segregated based on their morphological differences (Figure 1a). The lengths of the duodenum, jejunum, and ileum were found to be 9.6 ± 0.6 cm (*n* = 7 intestines), 26.0 ± 1.2 cm (*n* = 7 intestines), and 34.4 ± 1.7 cm (*n* = 5 intestines), respectively. These lengths are in agreement with previous reports.^22^ The jejunum was divided into three segments of equal length (~9 cm), denoted as proximal, medial, and distal jejunum (Figure 1b). The inner diameters of the intestinal segments were measured at the end of the duodenum, the beginning and end of the jejunum, and the beginning of the ileum (*n* = 3 intestines for each location; Figure 1c). The diameter at the end of the duodenum was 2.21 ± 0.04 mm, which was statistically similar to the diameters at the start (2.56 ± 0.31 mm) and end (2.60 ± 0.17 mm) of the jejunum. The diameter at the start of the ileum was 3.36 ± 0.07 mm, which was statistically higher (29%) than the diameter at end of the jejunum (*p* ≤ .05). Jejunum explants approximately 1 cm in length were excised and subsequently cultured in Transwell® inserts in 12‐well plates (Figure 1d,e).

### 2.2 Spatial differences in enterocyte markers

**Figure 2:**
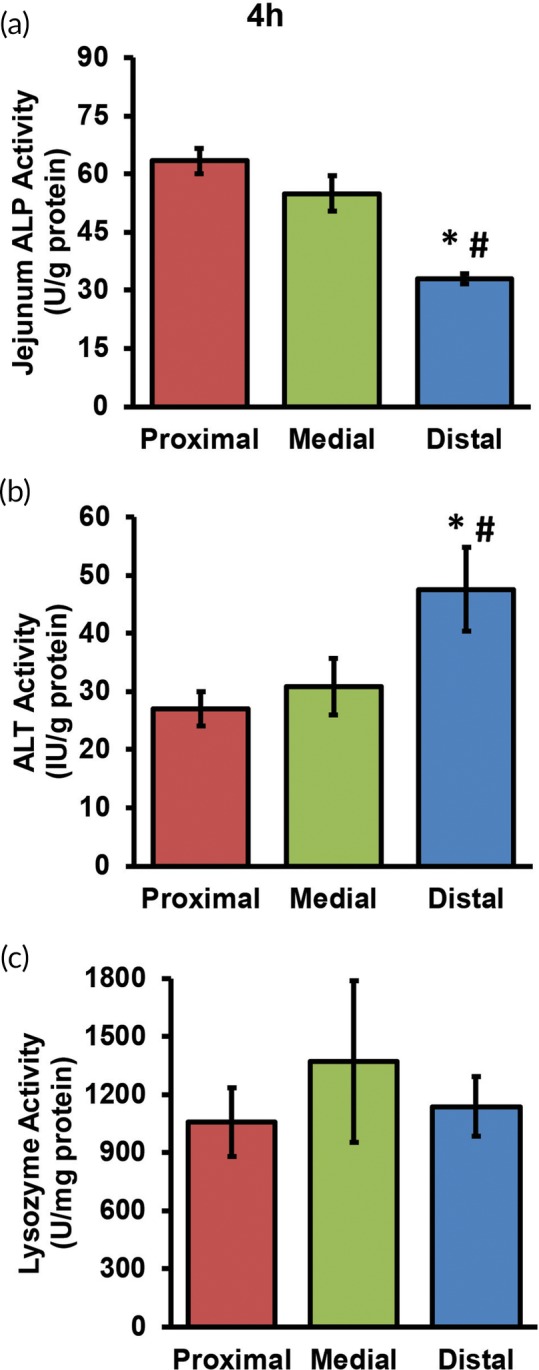
(a) Jejunum Alkaline Phosphatase (ALP) activity, (b) Alanine Aminotransferase (ALT) activity, and (c) lysozyme activity measured after 4 hr of explant culture. * and # denote *p* ≤ .05 relative to proximal and medial jejunal segments, respectively

Explants isolated from the jejunum were used to evaluate the spatial variation in enterocyte markers in the three jejunum locations (proximal, medial, and distal; *n* = 3 explants per location). ALP and ALT activities were measured after 4 and 24 hr of culture. At 4 hr, explants from the distal jejunum exhibited approximately twofold lower ALP activity (*p* ≤ .05) compared to values obtained from the proximal or medial sections. The ALP activities of the proximal and the medial sections were statistically similar (*p* > .05; Figure 2a). The decrease in ALP activity is consistent with reported in vivo observations in rats.^18^, ^21^ ALT is expressed in intestinal enterocytes and is involved in glutamine and glutamate metabolism.^23^ At 4 hr, the ALT activity in culture media from the distal jejunum was 1.8‐fold higher than in proximal and medial sections (*p* ≤ .05 for both proximal and medial comparisons; Figure 2b). However, at 24 hr, no significant differences were observed in the activities of three enzymes as a function of location (Figure S1A‐C). To evaluate enterocyte function at longer time points, ALP activity was measured after 72 hr in culture. Compared to the 24 hr time point, ALP activities of the proximal and medial jejunum decreased significantly at 72 hr (38 and 42%, respectively; *p* ≤ .05; Figure S2A). ALP activity in the distal segment decreased by 29% (*p* > .05).

### 2.3 Variations in Paneth cell function

Lysozyme activity was measured at 4 and 24 hr to assess Paneth cell function (*n* = 3 explants per jejunum location). The enzyme activity measured at 4 hr did not exhibit variation (Figure 2c). In contrast, a spatial trend was observed at 24 hr. Lysozyme activity increased significantly (40%) from proximal to distal jejunum (*p* ≤ .05; Figure S1c). Lysozyme activity in the medial jejunum was 43% higher than proximal, but this difference was not statistically significant (*p* > .05). Laval et al.^18^ reported an increase of approximately 2.5‐fold from proximal to distal jejunum in rats in vivo (0 hr), which is qualitatively similar to what is reported in the present study. Lysozyme activity was measured at 72 hr to evaluate Paneth cell function at longer time points. Lysozyme activity did not vary significantly (*p* > .05) compared to the activity at 24 hr for proximal, medial, or distal jejunum explants (Figure S2B).

### 2.4 Changes in epithelial cells and villi morphology

**Figure 3:**
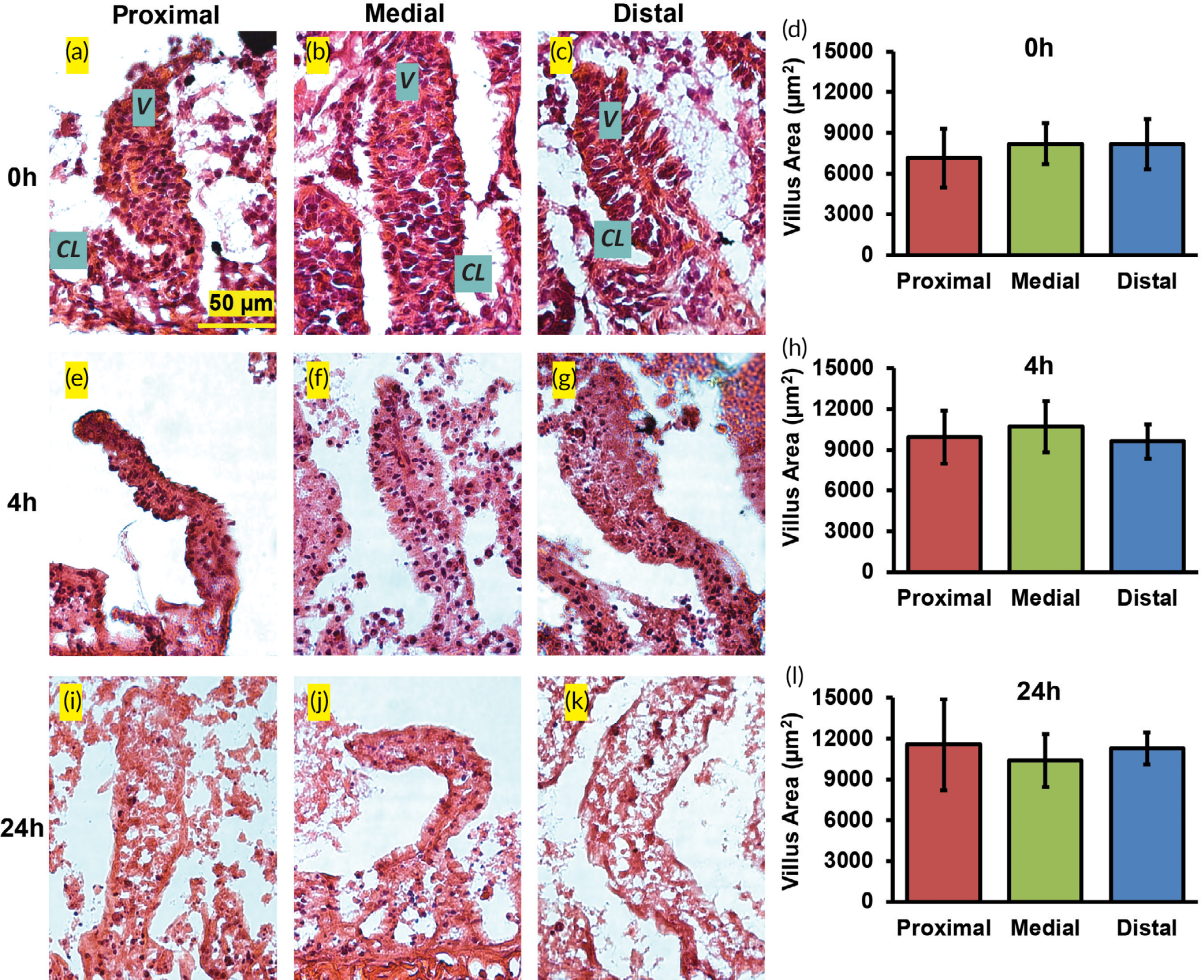
H&E staining of the proximal, medial, and distal jejunum after (a–c) 0 hr, (e–g) 4 hr, and (i–k) 24 hr in culture. V = villi, CL = crypts of Lieberkühn. Morphometric analysis of villus area across locations at (d) 0 hr, (h) 4 hr, and (l) 24 hr

H&E staining and villus area measurements were performed to investigate changes in morphology (Figure 3a–l). Explants from all three locations of the jejunum showed the crypt‐villus morphology at 0, 4, and 24 hr after culture as visualized through H&E staining (Figure 3a–c,e–g,i–k). The number of epithelial cells appeared to decrease with culture time with distal explants exhibiting the greatest decrease (Figure 3a–c,e–g,i–k). Villus area did not differ significantly between the proximal, medial and distal regions of the jejunum at the 0, 4, and 24 hr time points (*n* = 20 villi per condition; Figure 3d,h,l). Villus surface area has been reported to be similar among the duodenum, jejunum, and ileum in rats in vivo.^24^ Villus morphology appeared to be degraded in the distal jejunum after 72 hr in culture compared to the proximal and medial segments (Figure S2C–E). The villus area in the distal explants at 72 hr was significantly lower than the proximal or medial jejunum (42 and 38% lower, respectively; *n* = 20 villi per condition; *p* ≤ .05 for both; Figure S2F).

**Figure 4:**
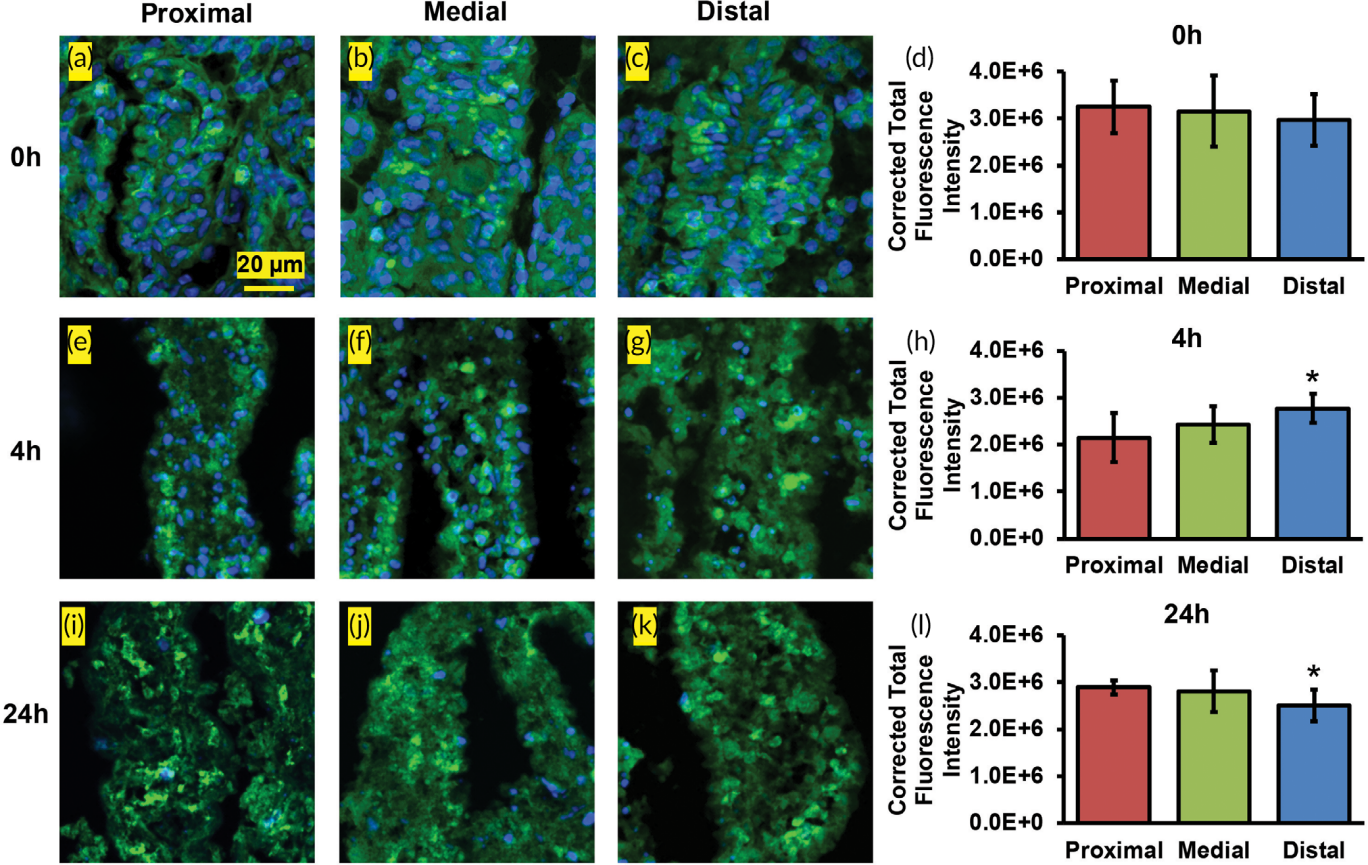
ZO‐1 immunofluorescence staining of the proximal, medial, and distal jejunum after (a–c) 0 hr, (e–g) 4 hr, and (i–k) 24 hr in culture. Green = ZO‐1 immunofluorescence, Blue = nuclei. Corrected total fluorescence intensity measurements at (d) 0 hr, (h) 4 hr, and (l) 24 hr

The expression of ZO‐1, a tight junction protein in the intestinal epithelium, was investigated by immunofluorescence staining. Corrected total fluorescence intensity of ZO‐1 expression in villi was measured using *n* = 10 images per condition. Differences were investigated across the three locations at the 0, 4, and 24 hr time points (spatial), and from one time point to the other within a location on the jejunum (temporal). Spatially, ZO‐1 immunofluorescence did not change significantly across locations at 0 hr (*p* > .05; Figure 4a–d). This is consistent with ZO‐1 trends reported in mice in vivo (0 hr) where the expression of ZO‐1 was unchanged across the length of the small intestine.^25^ However, at 4 hr, ZO‐1 immunofluorescence was 29% higher (*p* ≤ .05) in the distal jejunum compared to the proximal jejunum (Figure 4e–h). At 24 hr, the ZO‐1 immunofluorescence decreased by 14% (*p* ≤ .05) in the distal jejunum relative to the proximal jejunum (Figure 4i–l).

Temporal effects were also observed in ZO‐1 immunofluorescence. At 4 hr, ZO‐1 immunofluorescence decreased significantly in proximal jejunum (34%) compared to 0 hr (*p* ≤ .05 for both), while no significant change was observed in the medial and distal sections (*p* > .05). The decrease could be a result of loss of epithelial cells from the villi at later time points as observed by H&E staining. ZO‐1 immunofluorescence in explants from all three locations did not change significantly between 0 and 24 hr (*p* > .05). From 4 to 24 hr, the ZO‐1 intensity increased in proximal jejunum by 35% (*p* ≤ .05). However, no significant change in ZO‐1 immunofluorescence was observed in medial and distal explants between 4 and 24 hr (*p* > .05).

### 2.5 Goblet cell function

**Figure 5:**
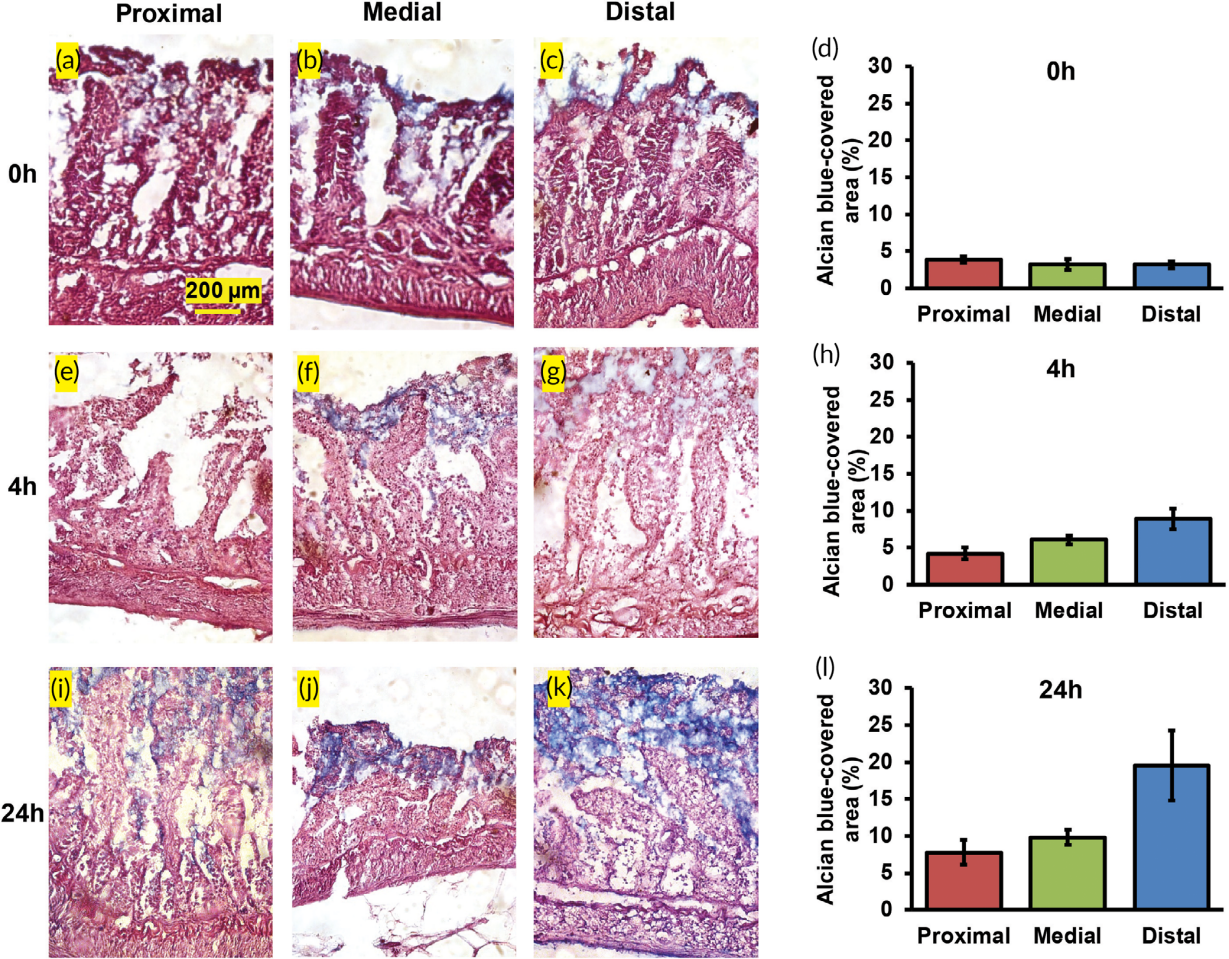
Alcian blue/PAS staining for mucins in the proximal, medial, and distal jejunum after (a–c) 0 hr, (e–g) 4 hr, and (i–k) 24 hr in culture. Alcian blue‐covered area fractions across locations at (d) 0 hr, (h) 4 hr, and (l) 24 hr

Goblet cell function was evaluated through AB/PAS staining of the explant samples.^26^ The area of an explant covered by mucin was measured as a function of location as well as time. Spatial and temporal variations are discussed individually. At 0 hr, mucins in the explants from all three locations covered only 3–4% of the cryosection area (Figure 5a–d). However, at 4 and 24 hr, the area of mucin‐covered regions increased for all three locations of the jejunum (Figure 5e–l). At 4 hr, the mucin‐covered area increased approximately twofold from the proximal to the distal jejunum, but this increase was not statistically significant (*p* > .05; Figure 5h). A ~1.5‐fold increase in the mucin‐covered area fraction was measured from the medial to the distal jejunum (*p* > .05). At 24 hr, the areas of mucin‐covered regions for the proximal and medial jejunum were similar (*p* > .05; Figure 5l). The distal jejunum exhibited approximately twofold higher mucin‐covered regions than proximal or medial explants (*p* > .05 for both; Figure 5l). In the rat intestine, an approximate 30–50% increase in the mucin volume from proximal to distal regions has been reported in vivo (0 hr).^19^ Although, our data may not be statistically significant, we do observe increases in the area of an explant covered by mucins.

Temporal variations were also observed within each location on the jejunum. In the proximal jejunum, the mucin‐covered area fractions at 0, 4, and 24 hr were 3.9 ± 0.4%, 4.2 ± 0.8%, and 7.8 ± 1.7%, respectively (*n* = 3 cryosections at each time point; Figure 5d,h,l). In the medial jejunum, the mucin‐covered area fractions at 0, 4, and 24 hr were 3.2 ± 0.8%, 6.0 ± 0.6%, and 9.8 ± 1.0%, respectively (*n* = 3 cryosections at each time point; Figure 5d,h,l). In the distal jejunum, the mucin‐covered area fractions at 0, 4, and 24 hr were 3.2 ± 0.5%, 8.9 ± 1.3%, and 19.5 ± 4.7%, respectively (*n* = 3 cryosections at each time point; Figure 5d,h,l).

In the proximal jejunum, the mucin‐covered area fraction at 24 hr was twofold higher than at 0 hr (*p* > .05). No significant change was observed in the mucin‐covered area at 72 hr compared to 24 hr (*p* > .05; Figure S2C,G). In the medial explants, the mucin‐covered area fractions increased significantly by threefold from 0 to 24 hr (*p* ≤ .05). At 72 hr, the mucin‐covered area fraction (9.8 ± 1.5%, *n* = 3) was similar to that at the 24 hr time point (*p* > .05; Figure S2D,G). In the distal explants, mucin‐covered area fractions increased significantly by 2.8‐fold from 0 to 4 hr and by sixfold from 0 to 24 hr (*p* ≤ .05 for both comparisons; Figure 5d,h,l). However, a 5.3‐fold decrease in mucin‐covered area was observed after 72 hr compared to 24 hr in culture (*p* ≤ .05; Figure S2E,G).

### 2.6 Integration of hepatocyte cultures and jejunum explants

**Figure 6:**
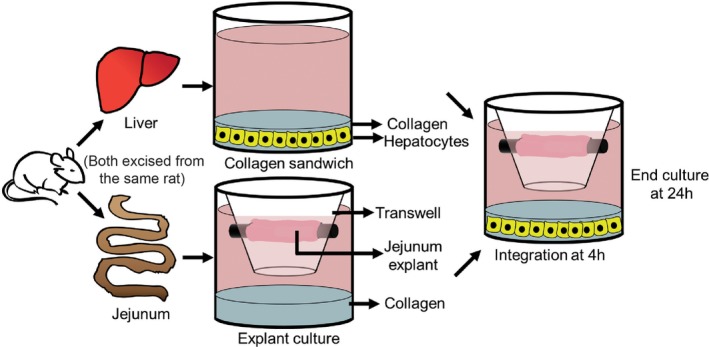
Schematic depicting the timeline for culture and integration of jejunum explants with hepatocyte collagen sandwich (CS) cultures. Jejunum explants and hepatocytes isolated from the same rat were integrated 4 hr postisolation and ended at the 24 hr time point

**Figure 7:**
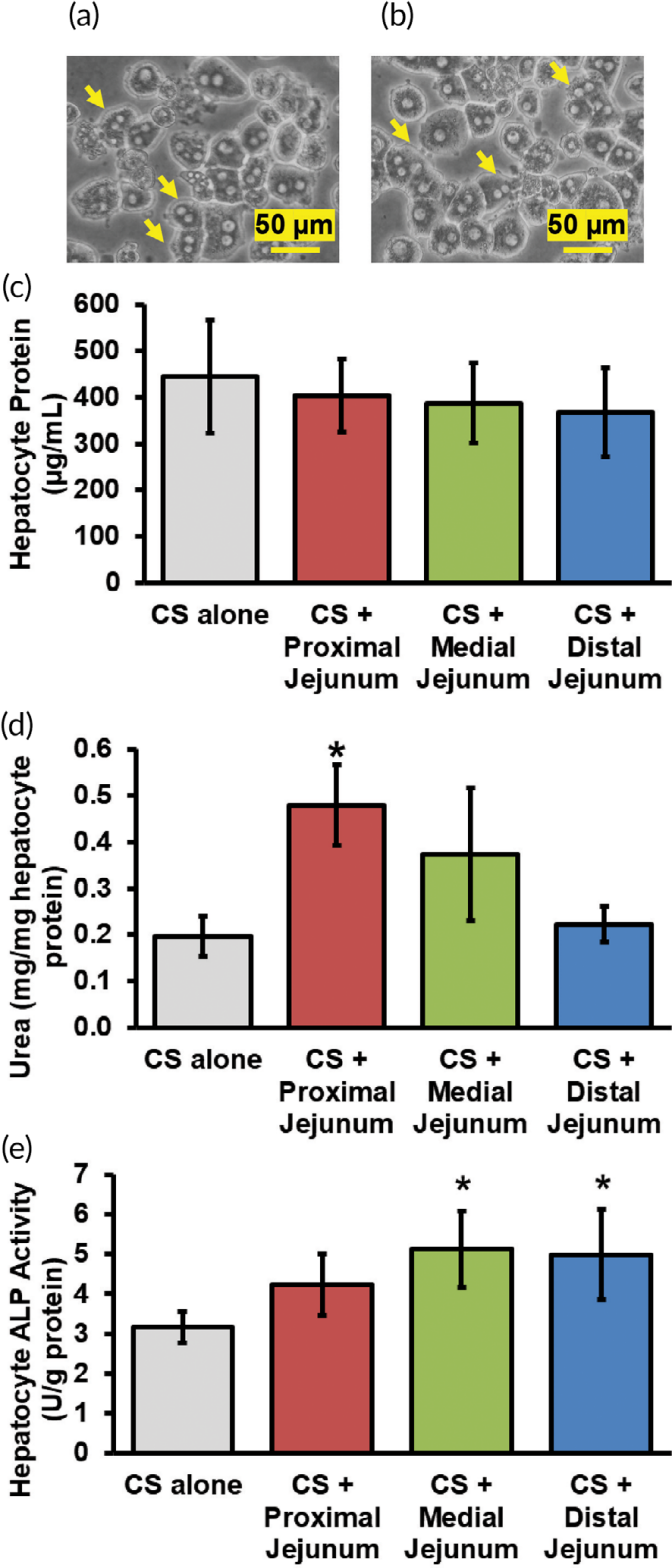
(a and b) Phase contrast images of hepatocytes in collagen sandwich cultures at the 24 hr time point. Arrows indicate binucleated hepatocytes. Effect of integration on hepatocytes. (c) Hepatocyte protein, (d) urea production, and (e) ALP activity in CS alone and integrated cultures (CS + proximal jejunum, CS + medial jejunum, and CS + distal jejunum). * denotes *p* ≤ .05 relative to CS alone cultures. CS, collagen sandwich

Jejunum explants were integrated with CS cultures of primary rat hepatocytes to understand how the two systems modulated each other's function. Both the jejunum and the hepatocytes were obtained from the same rat for the assembly of integrated intestinal‐hepatocyte cultures (Figure 6). Proximal, medial, and distal jejunum explants were integrated with hepatocytes and functions were evaluated 20 hr postintegration (Figure 7). Integrated cultures with proximal, medial, and distal jejunum explants are henceforth referred to as “proximal‐integrated,” “medial‐integrated,” and “distal‐integrated” cultures, respectively.

Phase contrast images (Figure 7a,b**)** and H&E staining (Figure S3) of hepatocytes in CS cultures exhibited binucleated cells with polygonal morphologies. The protein content of hepatocyte CS cultures did not change as a function of integration (Figure 7c) indicating that the jejunum did not cause an adverse effect on protein expression in hepatocytes. In proximal integrated cultures, urea secretion was ~2.4‐fold higher (*p* ≤ .05) than by hepatocytes cultured alone (Figure 7d). Medial and distal integrated cultures did not exhibit a statistically significant change (*p* > .05; Figure 7d). Hepatocytes in proximal integrated cultures did not exhibit a statistically significant change in ALP activity compared to CS alone controls (*p* > .05; Figure 7e). However, hepatocyte ALP activity increased significantly in medial (62%) and distal (58%) integrated cultures compared to CS alone controls (*p* ≤ .05 for both; Figure 7e). These trends indicate that integration with proximal jejunum explants may have improved hepatocyte function, while medial and distal jejunum explants may have a negative effect on hepatocytes.

**Figure 8:**
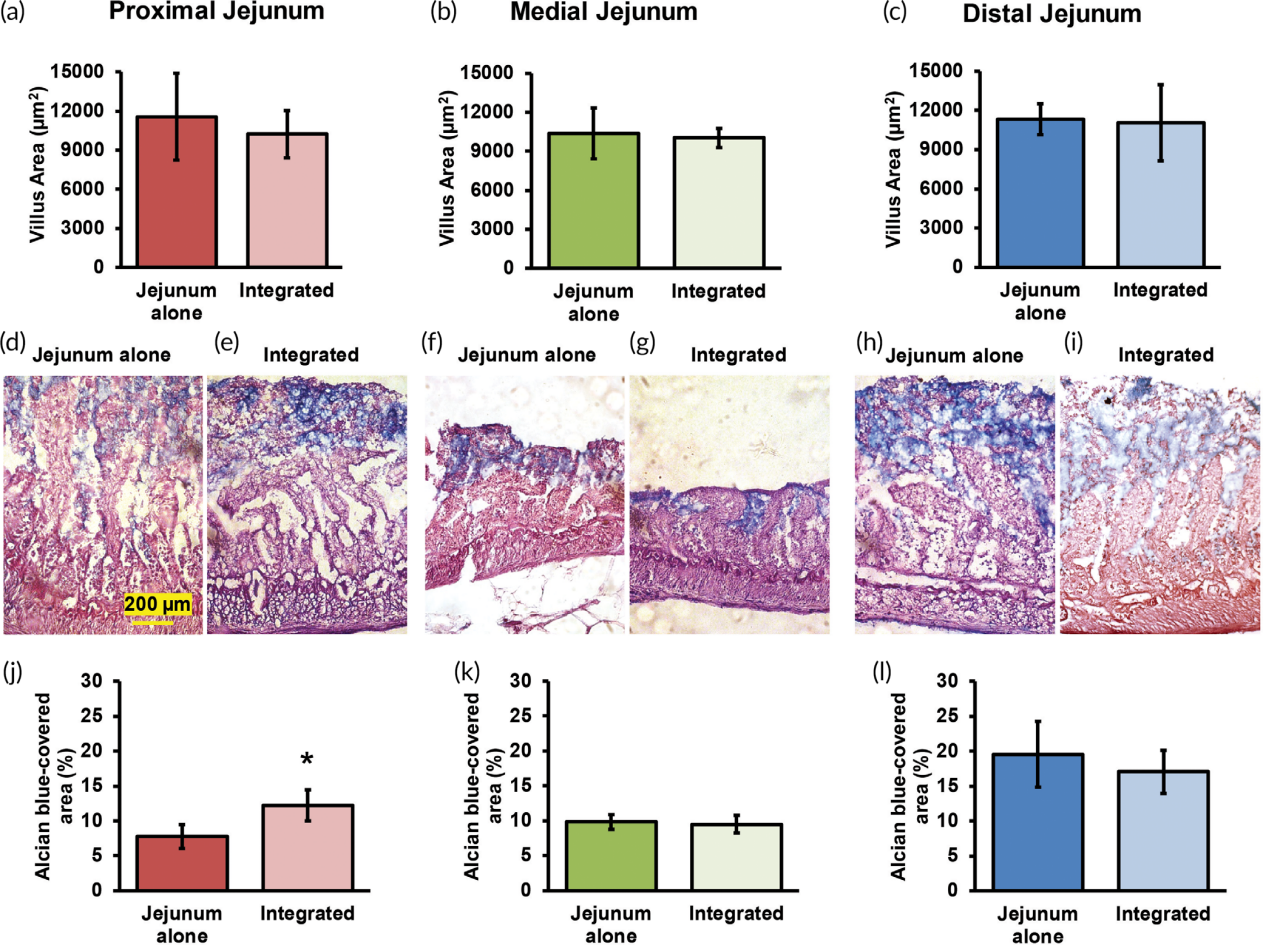
Effect of integration on jejunum explants. (a–c) Villus area and (d–i) Alcian blue/PAS staining of proximal, medial, and distal explants cultured alone or with hepatocyte collagen sandwich cultures. (j–l) Mucin‐covered area fractions in proximal, medial, and distal segments, respectively. * denotes *p* ≤ .05 relative to corresponding jejunum alone cultures

ALP and lysozyme activities in jejunum explants did not change significantly in proximal, medial, or distal integrated cultures, suggesting that there were no deleterious effects as a result of integration (Figure S4A–F). Changes in villus area upon integration were not statistically significant for proximal, medial, or distal jejunum (*p* > .05; Figure 8a–c). AB/PAS staining was performed to investigate the effect of integration of jejunum explants with hepatocytes on the mucus barrier (Figure 8d–i). AB/PAS staining revealed that the fraction of mucin‐covered cryosection area was significantly higher (57%) in proximal integrated cultures than the corresponding jejunum alone controls (*p* ≤ .05; Figure 8d,e,j). This result suggests an effect of hepatocytes on the proximal jejunum explants. This was not true for explants from the medial or distal jejunum (Figure 8f–i,k,l).

## 3 DISCUSSION

The small intestine exhibits variations in its absorptive, secretory, and metabolic functions based on location.^5^, ^18^, ^19^ To understand these spatial effects on intestinal function in culture, it is necessary to divide the intestine into small segments and separately investigate these sections in vitro. Most studies on the small intestine only report location‐dependent trends among the duodenum, the jejunum, and the ileum in vivo.^5^, ^18^, ^19^ For these reasons, we investigated the properties in different regions within the rat jejunum prior to integrating with hepatocytes.

Explants from the proximal, medial, and distal regions of the jejunum were cultured and functional and morphological differences between these regions were evaluated. ALP activity decreased from proximal to distal jejunum at 4 hr. Similar trends have been reported in the small intestine of rats.^18^, ^21^ Within the jejunum, one study reported that the ALP activity in the proximal end of the rat jejunum at 0 hr (in vivo) is approximately 1.6‐fold higher than that of the distal jejunum.^21^ At 24 hr, no significant differences were observed between the ALP activity among the three regions. This could be due to the presence of bacterial endotoxins such as lipopolysaccharides.^27^ Since intestinal bacteria increase from the proximal to the distal regions of the intestine,^26^ it may explain increased lysozyme secretion in the distal jejunum explants by 24 hr. Future studies will focus on identifying the specific endotoxins that can cause increased ALP activity and changes in bacterial growth as a function of jejunum location.

ALT activity in rat intestinal homogenates has been shown to increase from the stomach to the duodenum to the jejunum, followed by a decrease from jejunum to ileum.^28^ This suggests that ALT activity could increase along the length of the jejunum, as reported in the present study. ALT activity did not change significantly across the jejunum at 24 hr. One possible reason for this observation could be that enterocytes are continually shed from the tips of the villi^29^ and it is likely that at later time points, cell shedding affected the activity of this enzyme.

Further, spatiotemporal trends were revealed upon analyzing the area covered by the mucus barrier. Mucus deposition occurs over time and can take up to approximately 4.5 hr for its renewal.^30^ At 0 hr, the inversion process may have caused damage to the mucin layer, which may explain the location‐based differences starting after 4 hr in culture. It has been shown in vivo that the number of goblet cells increases along the length of the intestine from the duodenum to the distal ileum.^31^, ^32^ There were significant increases in mucin‐covered areas between 0 and 24 hr in explants obtained from the medial or distal jejunum sections. Although the increase in the area covered by mucins from proximal to medial to distal jejunum in the present study was similar to in vivo observations,^19^ these spatial trends were not statistically significant. Taken together, our measurements and observations demonstrate that the mucin barrier, an important intestinal characteristic is maintained up to 72 hr in culture in the proximal and medial regions of the jejunum, although, degradation was observed in the distal sections.

Temporal trends were observed in ZO‐1 expression in proximal jejunum explants. ZO‐1 immunofluorescence increased from 4 to 24 hr in proximal jejunum explants in culture. In the future, additional measurements such as western blots or ELISA assays will provide more comprehensive information.

We measured the functional markers for enterocyte, goblet, and Paneth cells in jejunum explants after 72 hr in culture to investigate the extent to which explants can be cultured in vitro. The change in ALP activity between 24 and 72 hr of culture in the proximal and medial jejunum suggests decreased enterocyte function. These results support previously reported observations of degradation of adult rat jejunum explants after 24 hr in culture.^8^ The trends in mucin‐covered area fractions between 24 and 72 hr could have resulted from decreased goblet cell function or the release of mucin proteins from the adherent mucus barrier into the cell culture media. Future investigations into specific mechanisms will provide information on which process causes such changes.

The second part of the study aimed to understand how individual cell functions could change when jejunum explants were integrated with hepatocyte CS cultures. To the best of our knowledge, only one study so far has described the integration of the intestine and the liver using primary cells and tissues.^12^ However, the focus of van Midwoud et al. was to investigate changes in Phase I and Phase II metabolism. Moreover, the two tissue slices were only integrated for 3–7 hr.

Since our data showed that the proximal, medial, and distal sections of the jejunum behaved differently from each other in culture, we maintained the distinction between these regions of the jejunum for our investigations with integrated jejunum‐hepatocyte cultures as well. The integration of hepatocyte CS cultures with explants from the proximal, medial, and distal jejunum did not affect hepatocyte viability, or enterocyte and Paneth cells functions significantly. However, an effect of explant location was observed in urea secretion and hepatocyte ALP activity. Urea secretion was significantly higher in hepatocytes integrated with proximal jejunum explants. *In vivo*, the small intestine produces ammonia, which is detoxified by conversion to urea in the liver.^6^ Future studies will focus upon measuring changes in ammonia to correlate to variations in urea concentration.^6^ Hepatocyte ALP activity was significantly increased in medial and distal integrated cultures. Since bacterial endotoxins may have been the reason for this trend, our future studies will focus upon identifying specific toxins that may have played a role.

## 4 CONCLUSIONS

The spatiotemporal trends obtained in jejunum explants suggest that cellular and morphological markers change even within this region of the intestine. The trends reported demonstrate the importance of knowing which region of the jejunum is used when conducting future investigations into metabolism or biotransformation. The intestine‐hepatocyte models described and evaluated in this study will be very useful for studies on understanding the crosstalk between these organs, specifically in the areas of drug discovery, validation, and in their biotransformation.

## 5 MATERIALS AND METHODS

Alcian blue, calcium chloride, collagenase Type IV, gentamicin sulfate, glucagon, glutaraldehyde, 4‐(2‐hydroxyethyl)‐piperazine‐1‐ethanesulfonic acid, hydrocortisone, periodic acid, protease inhibitor cocktail, and Schiff's reagent were purchased from Sigma‐Aldrich (St. Louis, MO). All other chemicals were purchased from Thermo Fisher Scientific (Waltham, MA) unless otherwise stated.

### 5.1 Isolation and culture of primary rat hepatocytes

Primary hepatocytes were isolated from female Lewis rats weighing 180–210 g (Envigo, Indianapolis, IN) using an in situ two‐step collagenase method as previously described before.^33^, ^34^ Animal care and excision protocols were approved by and conducted in accordance with the Virginia Tech Institutional Animal Care and Use Committee. Total isolated hepatocyte counts typically lay in the range of 100–150 million hepatocytes with ≥97% viability, as determined by trypan blue exclusion. Hepatocytes were seeded on collagen gels at a cell density of 5 x 10^5^ cells/well in a 12‐well plate. A second collagen gel layer (1.1 mg/ml) was deposited on top of the hepatocyte monolayer approximately 3 hr post‐hepatocyte seeding, to assemble CS hepatocyte cultures. Hepatocyte culture medium was added 4 hr postseeding. Hepatocyte culture medium was composed of Dulbecco's modified Eagle medium, with 0.37% sodium bicarbonate, 10% (vol/vol) heat‐inactivated fetal bovine serum, 200 U/ml penicillin, 200 μg/ml streptomycin, 0.5 U/ml insulin (MP Biomedicals, Santa Ana, CA), 20 ng/ml epidermal growth factor, 14.28 ng/ml glucagon, and 7.65 μg/ml hydrocortisone. Cultures were maintained at 37°C in a humidified incubator with 10% carbon dioxide.

### 5.2 Isolation and culture of rat jejunum explants

The small intestine was excised from female Lewis rats from which the liver was excised. The duodenum was removed at the ligament of Treitz (Figure 1a). The jejunum was separated from the ileum at the zone where there was an increase in the diameter and amount of mesenchyme (Figure 1c). A detailed procedure on the excision of jejunum explants has been previously reported.^10^ Briefly, an inverted jejunum segment (approximately 1 cm in length) was pulled on Matrigel®‐coated polydimethylsiloxane rods, placed in a Millicell® insert (Figure 1d,e) and transferred to 12‐well plates containing collagen gels. Oxygenated gentamicin‐containing medium (3 ml) was added to each well. Each jejunum segment resulted in approximately 18 explants, with an average length of 9.9 ± 0.4 mm (*n* = 84 explants from seven rats; Figure 1d). The top six sections were identified as proximal, the middle six as medial, and the bottom six as distal (Figure 1b). Explant cultures were maintained at 37°C in a humidified incubator with 10% CO_2_. The media was removed and replenished at 3 and 4 hr. At 4 hr, the explants were transferred to new collagen‐coated wells to be maintained as jejunum cultures, or to well‐plates that contained hepatocyte cultures to assemble integrated jejunum‐hepatocyte cultures. All cultures (CS alone, jejunum, or integrated) were supplemented with 3 ml of hepatocyte medium.

### 5.3 Measurement of alkaline phosphatase activity

ALP activity was measured using a commercially obtained assay kit (Abcam, Cambridge, UK) following the manufacturer's instructions. Briefly, jejunum explant lysates and hepatocyte lysates were incubated with para‐nitrophenyl phosphate (pNPP) substrate for 1 hr at 25°C, following which the absorbance was measured at 405 nm to quantify the para‐nitrophenol (pNP) produced through ALP‐mediated dephosphorylation. A standard curve was generated using the pNPP substrate and ALP enzyme provided by the manufacturer.

$$\mathrm{ALP}\;\text{activity}\;\left(\frac{U}{\mathrm{ml}}\right)=\frac{\frac{A}{V}}{T},$$

where *A* = concentration of pNP generated in samples (μmol), *V* = volume of sample (ml), *T* = reaction time (min). ALP activity values were normalized to the protein content of each sample.

### 5.4 Measurement of lysozyme activity

Lysozyme activity in spent culture media was measured using a commercially available assay kit (EnzChek™ Lysozyme Assay Kit, Thermo Fisher Scientific) following the manufacturer's protocol. Briefly, spent culture media was incubated with fluorescein‐conjugated *Micrococcus lysodeikticus* cell walls that acted as a lysozyme substrate. Fluorescence was measured after 2 hr of incubation at 37°C at excitation and emission wavelengths of 485 and 538 nm, respectively. Lysozyme activity values were calculated through a standard curve and normalized to protein content.

### 5.5 Statistical analyses

Statistical significance was determined using *p*‐values determined by one‐tailed Student's *t* tests, assuming unequal variance when sample sizes were unequal, and equal variance when sample sizes were equal between the groups being compared. The Bonferroni correction (multiple hypothesis testing) was applied with *α* = 0.05. All data are reported as mean ± standard deviation, and *n* denotes sample size.

## CONFLICT OF INTEREST

The authors have no conflict of interest to declare.

## Supporting information


**Data S1**: Supplementary information
