# Supplementary material for: The assembly of integrated rat intestinal‐hepatocyte cultures
Source: Bioeng Transl Med. 2019 Nov 9;5(1):e10146. doi: 10.1002/btm2.10146 (PMC6971435; doi:10.1002/btm2.10146)
Supplement: Supplementary file 1 — Data S1: Supplementary information [file BTM2-5-e10146-s001.docx]

**SUPPLEMENTARY INFORMATION**

**The Assembly of Integrated Rat Intestinal-Hepatocyte Cultures**

*Anjaney Kothari, ^1^ and Padmavathy Rajagopalan, ^1, 2, 3, *^*

1. School of Biomedical Engineering and Sciences, Virginia Tech, Blacksburg, VA 24061, United States

2. Department of Chemical Engineering, Virginia Tech, Blacksburg, VA 24061, United States

3. ICTAS Center for Systems Biology of Engineered Tissues, Virginia Tech, Blacksburg, VA 24061, United States

***Corresponding Author Information:**

Prof. Padmavathy Rajagopalan

Department of Chemical Engineering

Virginia Tech, Blacksburg VA 24061

Email: padmar@vt.edu, Tel: +1-540-231-4851

**SUPPLEMENTARY METHODS**

***Collagen extraction***

Type I collagen was extracted from rat tails as described previously ^1,2^. Briefly, rat tail tendons were dissolved in acetic acid. The resulting solution was centrifuged at 13,000 x *g*, following which collagen was precipitated with 30% (w/v) sodium chloride. Precipitated collagen was centrifuged at 8,500 x *g* and the pellets were resuspended in 0.6% acetic acid. The resulting suspension was dialyzed against 1 mN hydrochloric acid. Collagen concentration was measured by measuring absorbance at 280 nm and used at a pH of 3.1. Collagen was sterilized with chloroform prior to cell culture. Collagen gels were cast at a concentration of 1.1 mg/mL in 12-well plates (250 µL per well for hepatocyte seeding and 500 µL per well for jejunum explant cultures).

***Protein measurements in hepatocyte cultures and jejunum explants***

All CS cultures were ended through collagenase IV-mediated digestion of the collagen gels. This was followed by the lysis of hepatocyte protein in the presence of a protease inhibitor cocktail. The protease inhibitor cocktail consisted of 4-(2-aminoethyl) benzenesulfonyl fluoride hydrochloride, aprotinin, bestatin, E-64, leupeptin, and pepstatin A as described before ^1^. Jejunum explants were homogenized using a TissueRuptor® homogenizer (Qiagen, Hilden, Germany) at a speed setting of 8 for 2 min per sample. Homogenization was performed in 0.5 mL lysis buffer in presence of the protease inhibitor cocktail to isolate protein. Homogenates were centrifuged at 10,000x *g* for 5 min and the supernatant was collected and frozen until further use. Protein concentrations in cell or tissue lysates were determined through the Bradford assay using a commercially available kit (Coomassie (Bradford) Protein Assay Kit; Thermo Fisher Scientific) as previously described ^1^.

***Measurement of alanine aminotransferase (ALT) activity***

Alanine aminotransferase (ALT) activity was measured using a commercially obtained kit (ALT (SGPT) Reagent, Colorimetric, Endpoint Method; Teco Diagnostics, Anaheim, CA) following the manufacturer’s protocol ^1^. The absorbance was measured at 505 nm. Absorbance values were converted to enzyme concentrations using a calibrator of known ALT activity provided by the manufacturer.

***Urea Secretion***

Urea secretion was measured as described previously ^2,3^ through a colorimetric, diacetyl monoxime based assay using a commercially available Blood Urea Nitrogen (BUN) assay kit (Stanbio Laboratory, Boerne, TX). Absorbance was measured at 520 nm and a standard curve was generated using urea diluted in culture medium.

***Cryosectioning of jejunum explants***

Explants were cut lengthwise and fixed with 2 mL of glutaraldehyde (3% w/v) at 4°C for 7h. Fixed samples were washed with PBS (1X), followed by a 5 min incubation in 15% (w/v) sucrose, and a 15 min incubation in 30% (w/v) sucrose solutions. Thereafter, samples were equilibrated in Tissue-Tek Optimal Cutting Temperature (OCT) compound (Electron Microscopy Sciences, Hatfield, PA) for 15 min. Samples were frozen in OCT compound in cryomolds using an isopentane solution partially frozen with liquid nitrogen ^4^. The embedded samples were then cryosectioned transversely at a thickness of 10 µm per section to visualize the intestine. Sections were stored at -80°C until used. Prior to histochemical or immunofluorescence staining, cryosections were fixed with 3% (w/v) glutaraldehyde (in PBS (1X)) for 20 min at room temperature.

***Hematoxylin and Eosin staining of jejunum explants***

Jejunum cryosections were stained with Mayer’s Hematoxylin and Eosin according to the manufacturer’s instructions ^5^. Briefly, cryosections were stained with Mayer’s hematoxylin solution, and rinsed in warm water (pH = 8.2-8.4). Thereafter, sections were dehydrated in 95% ethanol and stained with Eosin Y solution (0.5% (w/v), in 95% (v/v) ethanol alcoholic solution, pH = 4.2 - 4.3). Stained sections were decolorized in 95% ethanol until streaking of the eosin stain ceased. Finally, samples were serially dehydrated for 2 min each in 95% (v/v) ethanol and absolute ethanol and mounted on glass slides with Cytoseal™ 60.

***Alcian blue (AB)/Periodic acid-Schiff’s base (PAS) staining of jejunum explants***

Jejunum cryosections were stained with Alcian blue/PAS staining to visualize acidic and neutral mucins. Staining procedures were adapted from a previous report ^6^. Briefly, samples were equilibrated with 3% (v/v) acetic acid and incubated with the Alcian blue dye (1% (w/v) in 3% (v/v) acetic acid, pH = 2.5) for 2.5h. Non-specific stain was removed with 3% (v/v) acetic acid. Thereafter, samples were incubated in 0.1% (w/v) periodic acid, followed by incubation with Schiff’s reagent. Stained samples were washed in running tap water for 10 min, rinsed in deionized water, and serially dehydrated for 2 min each in 95% (v/v) ethanol and absolute ethanol. Finally, the samples were mounted on glass slides with Cytoseal™ 60.

***Morphometric analyses***

Villus area was measured using FIJI (ImageJ software, NIH) ^7^ for n = 20 villi per condition. The fraction of each cryosection stained with Alcian Blue was measured using FIJI (n = 3 cryosections per condition). Images of complete cryosections were thresholded using the hue, saturation and brightness (HSB) color model. The image hue and saturation parameters were adjusted to isolate blue-colored areas stained by AB in the samples. The blue colored area was then divided by total area of the cryosection to calculate the ‘mucin-covered area fraction’.

***Immunofluorescence staining for ZO-1***

Fixed samples were treated with 0.1% (w/v) sodium borohydride, followed by a 5 min incubation in 0.05% (v/v) TritonX-100. The samples were rinsed with warm PBS and blocked with 1% (w/v) BSA in PBS (1X) supplemented with rabbit serum (15µL/mL) at 37°C for 1h. Samples were incubated at 4°C overnight with primary monoclonal anti-rat ZO-1 antibody (Developmental Studies Hybridoma Bank, University of Iowa; 1:5 dilution). A FITC-conjugated rabbit anti-rat IgG secondary antibody was then used to visualize ZO-1 expression. Nuclei were stained with Hoechst 33258 dye. Samples were imaged using a Zeiss LSM confocal microscope (Oberkochen, Germany). Immunofluorescence was quantified through ImageJ as corrected total fluorescence intensity using n = 10 images as described previously ^8^. Corrected total fluorescence intensity = [Integrated fluorescent intensity] – [fluorescent area x mean background fluorescence]. The mean background fluorescence was measured as the mean of fluorescence intensities at 10 locations per image not corresponding to the villi.

**SUPPLEMENTARY DATA**

***Supplementary Figure 1***: (A) Jejunum ALP activity, (B) ALT activity and (C) lysozyme activity at 24h of culture.


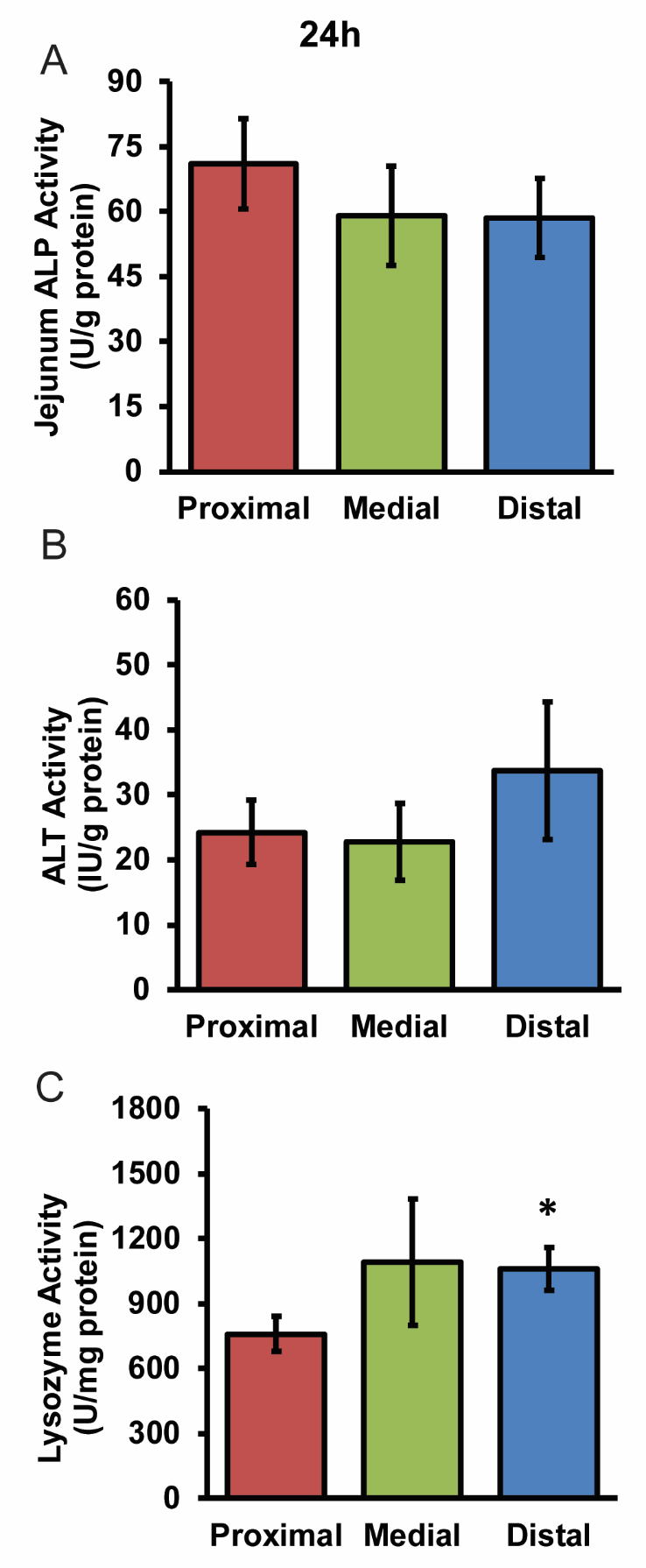


***Supplementary Figure 2***: (A) Jejunum ALP activity and (B) lysozyme activity at 24h and 72h after culture (* denotes *p* ≤ 0.05 relative to 24h time-point). Alcian blue/PAS staining for mucins in the (C) proximal, (D) medial and (E) distal jejunum after 72h in culture. (F) Villus areas and (G) alcian blue-covered area fractions across locations at 72h (* and # denote *p* ≤ 0.05 relative to proximal and medial jejunal segments, respectively).


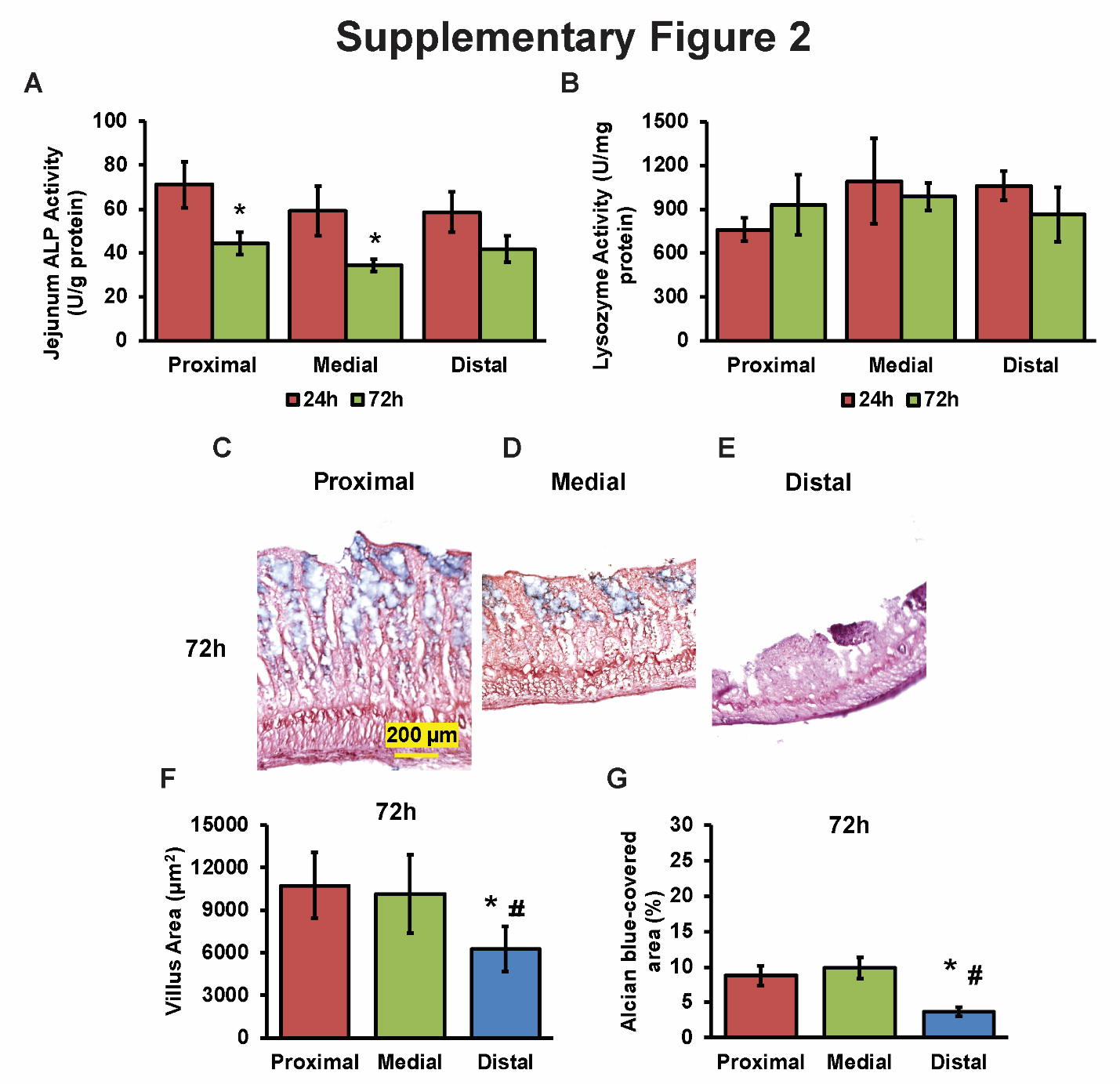


***Supplementary Figure 3***: Hematoxylin and Eosin staining of hepatocytes in CS cultures. Arrows indicate binucleated hepatocytes.


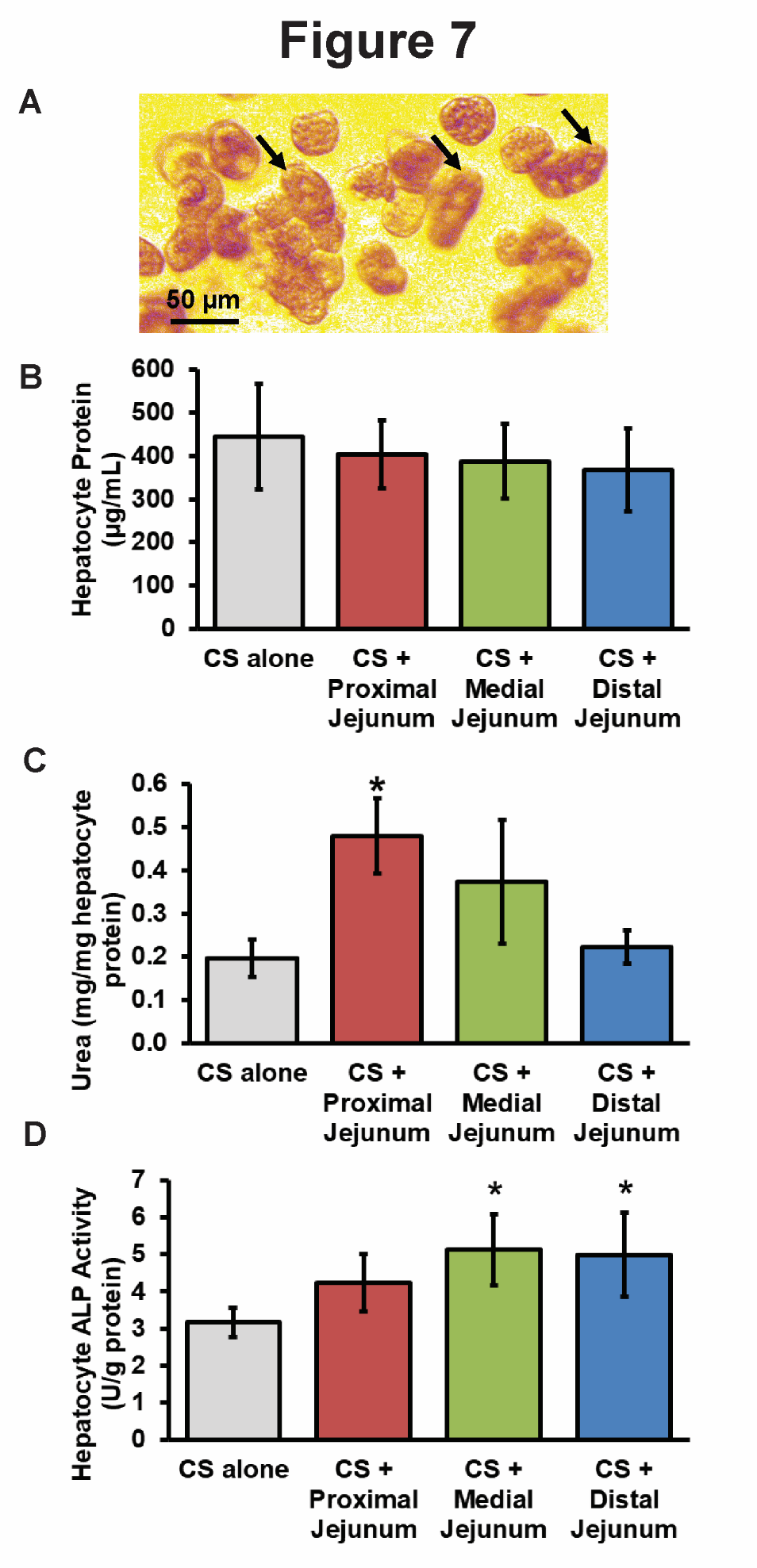


***Supplementary Figure 4***: Effects of integration on (A - C) enterocyte and (D – F) Paneth cell markers.


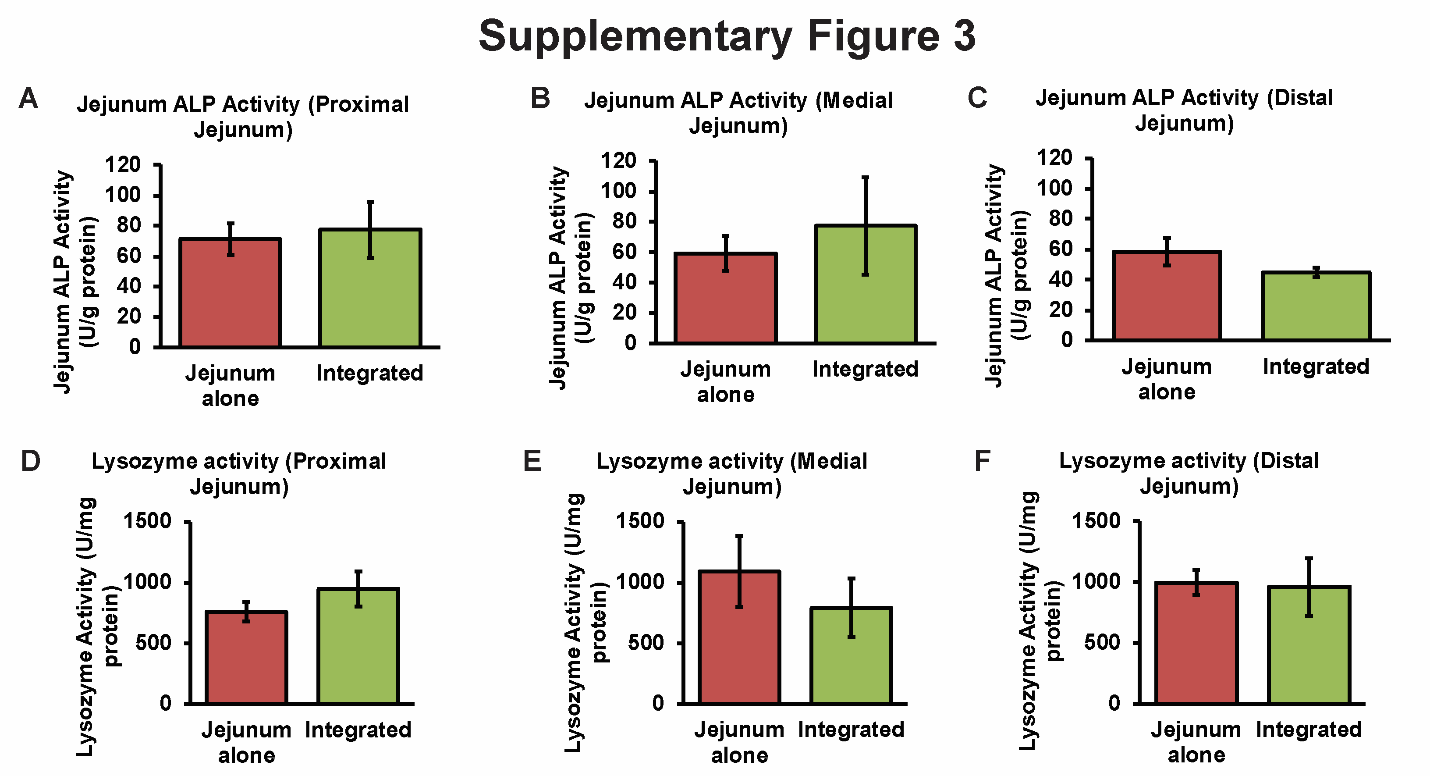


**References**

1. Orbach SM, Cassin ME, Ehrich MF, Rajagopalan P. Investigating acetaminophen hepatotoxicity in multi-cellular organotypic liver models. *Toxicol In Vitro.* 2017;42:10-20.

2. Orbach SM, Ehrich MF, Rajagopalan P. High-throughput toxicity testing of chemicals and mixtures in organotypic multi-cellular cultures of primary human hepatic cells. *Toxicol In Vitro.* 2018;51:83-94.

3. Kim Y, Larkin AL, Davis RM, Rajagopalan P. The design of in vitro liver sinusoid mimics using chitosan–hyaluronic acid polyelectrolyte multilayers. *Tissue Eng Part A.* 2010;16(9):2731-2741.

4. De Jesus M, Ahlawat S, Mantis NJ. Isolating and immunostaining lymphocytes and dendritic cells from murine Peyer's patches. *J Vis Exp.* 2013;73(73):e50167.

5. Fischer AH, Jacobson KA, Rose J, Zeller R. Hematoxylin and eosin staining of tissue and cell sections. *CSH Protoc.* 2008;2008(5):pdb. prot4986.

6. Cohen M, Varki NM, Jankowski MD, Gagneux P. Using unfixed, frozen tissues to study natural mucin distribution. *J Vis Exp.* 2012;67(67):e3928.

7. Schindelin J, Arganda-Carreras I, Frise E, et al. Fiji: an open-source platform for biological-image analysis. *Nat Methods.* 2012;9(7):676.

8. Ford AJ, Orbach SM, Rajagopalan P. Fibroblasts stimulate macrophage migration in interconnected extracellular matrices through tunnel formation and fiber alignment. *Biomaterials.* 2019;209:88-102.
